# TESTING AN INTERVENTION TO PREVENT CARETAKER NEGLECT: CHALLENGES IN RECRUITING STUDY PARTICIPANTS

**DOI:** 10.1093/geroni/igad104.0454

**Published:** 2023-12-21

**Authors:** Farida Ejaz, Samantha Tuft, Courtney Reynolds, Jessica Bibbo

**Affiliations:** Benjamin Rose Institute on Aging, Cleveland, Ohio, United States; Benjamin Rose Institute on Aging, Cleveland, Ohio, United States; Benjamin Rose Institute on Aging, Cleveland, Ohio, United States; Benjamin Rose Institute on Aging, Cleveland, Ohio, United States

## Abstract

Researchers collaborated with Utah Adult Protective Services (APS) to develop a telephone-based case management intervention to follow-up with both alleged victims and perpetrators of caretaker neglect after their case was determined to be inconclusive and closed by APS. We focused on this group because APS administrative data suggested that reoccurrence of caretaker neglect was common among this cohort. We hypothesized that linking both victims and caretakers with needed community-based services could prevent reoccurrence of caretaker neglect reports over time. In this presentation, we will describe the challenges we faced with recruiting alleged victims and perpetrators of caretaker neglect. In the piloting phase, we approached 87 potential participants; 18 did not meet study criteria, and 35 did not answer or had incorrect or non-working phone numbers. Of the remaining 34, 9 (26%) agreed to participate and one withdrew later during the follow-up phase. Interviewers reported that refusals were related to reading a 6-minute-long consent form over the phone and conducting an hour-long interview that included the research questionnaire and case-management intervention. The feedback led investigators to simplify the study design/protocols and to shorten the questionnaire to focus on critical data elements and the service needs of participants. Data collection is expected to continue across Utah until spring 2024. We will have a clearer sense of whether such adaptations increased response rates. In addition, we learned that research and practice/intervention goals are likely to be different but negotiating trade-offs, and establishing contracts and data-use agreements early on are important for successful partnerships.

